# Effectiveness of Combined Strategies for the Prevention of Hypothermia Measured by Noninvasive Zero-Heat Flux Thermometer During Cesarean Section

**DOI:** 10.3389/fmed.2021.734768

**Published:** 2021-12-23

**Authors:** Antonella Cotoia, Paola Sara Mariotti, Claudia Ferialdi, Pasquale Del Vecchio, Renata Beck, Simona Zaami, Gilda Cinnella

**Affiliations:** ^1^Department of Medical and Surgical Sciences, Anesthesia and Intensive Care Unit, University of Foggia, Foggia, Italy; ^2^Department of Anatomical, Histological, Forensic, and Orthopedic Science, Sapienza University of Rome, Rome, Italy

**Keywords:** spinal anesthesia, core temperature, perioperative hypothermia, SpotOn, cesarean delivery (CD)

## Abstract

**Background:** Perioperative hypothermia (body temperature <36°C) is a common complication of anesthesia increasing the risk for maternal cardiovascular events and coagulative disorders, and can also influence neonatal health. The aim of our work was to evaluate the impact of combined warming strategies on maternal core temperature, measured with the SpotOn. We hypothesized that combined modalities of active warming prevent hypothermia in pregnant women undergoing cesarean delivery with spinal anesthesia.

**Methods:** Seventy-eight pregnant women were randomly allocated into three study groups receiving warmed IV fluids and forced-air warming (AW), warmed IV fluids (WF), or no warming (NW). Noninvasive core temperature device (SpotOn) measured maternal core temperature intraoperatively and for 30 min after surgery. Maternal mean arterial pressure, incidence of shivering, thermal comfort and newborn's APGAR, axillary temperature, weight, and blood gas analysis were also recorded.

**Results:** Incidence of hypothermia was of 0% in AW, 4% in WF, and 47% in NW. Core temperature in AW was constantly higher than WF and NW groups. Incidence of shivering in perioperative time was significantly lower in AW and WF groups compared with the NW group (*p* < 0.04). Thermal comfort was higher in both AW and WF groups compared with NW group (*p* = 0.02 and *p* = 0.008, respectively). There were no significant differences among groups for the other evaluated parameters.

**Conclusion:** Combined modalities of active warming are effective in preventing perioperative hypothermia. The routine uses of combined AW are suggested in the setting of cesarean delivery.

## Introduction

The inadvertent perioperative hypothermia (PH) is the unintentional cooling of a patient's core temperature (CT°) >36°C (1).

Perioperative hypothermia is estimated to occur in >60% of patients receiving spinal anesthesia for cesarean delivery (2–4), in whom it significantly impairs thermal autoregulation by inhibiting the vasomotor and shivering responses even above the level of the sensory block and causes a thermal redistribution of heat from core to peripheral tissues (5–9).

In absence of strategies for preserving normothermia, patients become hypothermic in the early 30–40 min of surgery, and they remain hypothermic during postoperative time (10).

Hypothermia increases the risk of cardiovascular events, such as myocardial ischemia, arrhythmias, and coagulative disorders, greater blood loss with a need of transfusions, wounds infection with delayed healing due to decreased antibody- and cell-mediated immune responses, and also oxygen availability in the peripheral wound tissues. PH changes the kinetics and action of various anesthetic and paralyzing agents, increases thermal discomfort, and is associated with delayed postanesthetic recovery (2, 11–14).

Moreover, PH can increase oxygen consumption and catecholamines release, which are responsible of intraoperative complications, such as hypoxia and increased pulmonary vascular resistances, and postoperative shivering can increase the metabolic rate leading to an increased incidence of early postoperative myocardial ischemia, especially in high risk-patients (15, 16).

Maternal hypothermia during cesarean delivery can also influence the APGAR score of newborns, neonatal temperature, umbilical vein blood gas analysis (BGA), and blood coagulation (13). Those consequences are particularly relevant in light of the fact that failure to identify and treat perioperative hypothermia is starting to be acknowledged as a potential cause of malpractice lawsuits, in case adverse outcomes arise. It is worth bearing in mind that if a claim should be filed based on alleged medical malpractice stemming from negligence, courts (particularly under tort law statutes) are likely to hold doctors and facilities liable, if the patient medical records and informed consent documentation process turn out to be inaccurate or lacking in any measure. Such inconsistencies may be viewed by the court as resulting from negligent practices rather than unavoidable clinical complications. Overall, any failure to comply with surgical safety protocols or to produce documentation proving adherence to guidelines issued by recognized scientific societies and bodies, and therefore deemed reliable, will likely result in unfavorable rulings for doctors and hospitals (17–19).

Core temperature is the best single indicator of thermal status in humans. There are several methods to detect the body CT°, including: noninvasive, such as tympanic thermometer and rectal probe; and invasive, such as pulmonary artery catheter (PAC), esophageal and nasopharyngeal probes. All the studies on perioperative hypothermia used noninvasive CT° monitoring devices, especially tympanic thermometers.

A new skin temperature monitoring system, the SpotOn, measures the heat flux in a temporal artery and is a very reliable device in comparison with PAC, which is the gold standard.

The warming systems currently used to prevent and treat hypothermia, are classified into passive and active. Among the active warming methods, the most used are heated infusion fluids and forced air-warming systems; the latter can be applied on superior and/or lower extremities.

The multimodal approach of combined warming modalities (intravenous fluid infusion and forced air warming), temperature measurements, type of neuraxial anesthesia, duration, and time of warming (preoperative, preanesthetic, intraoperative) (2, 20, 21) have provided a series of interesting studies, but with no total consensus on the best management of perioperative hypothermia (2, 11, 12, 22).

Multiple studies used a single-modality warming intervention (warmed IV fluids (WF) or forced-air warming alone) and have shown little efficacy in preventing perioperative hypothermia during cesarean delivery (4, 20, 21, 23–27).

The aim of our prospective randomized controlled study is to evaluate the impact of combined strategies of warming in pregnant women undergoing cesarean delivery in terms of maternal CT° using the SpotOn.

## Methods

### Study Design and Patient Population

After ethical approval, our prospective randomized, controlled study was performed at the Obstetrics and Gynecology Department of the University Hospital of Foggia, Italy (ClinicalTrials.gov registration number: NCT03473470).

Consecutive patients with the following inclusion criteria were considered for enrollment: healthy pregnant women (ASA 1-2), age from 18 to 40 years, at term gestation (≥ 37 weeks), and elective cesarean delivery with spinal anesthesia. Exclusion criteria included conditions, such as fever, diabetes mellitus, BMI > 40 kg/m, coagulative disorders, preeclampsia, and all those factors that can cause intraoperative bleeding (placental abruption or history of placenta previa). Before data collection, the purpose of the work was carefully explained and a written informed consent was obtained from each participant, according to the Helsinki declaration. From the original sample of 93 pregnant women, only 78 were enrolled in the work.

Women were randomly assigned, by a computer-generated randomization, to three study groups (26 patients each) to receive WF alone, warmed IV fluids and forced-air warming (AW), or IV fluids at room temperature with further no warming (NW).

### Study Protocol

Demographic data, vital signs (heart rate, mean arterial pressure, and pulse oximetry), and CT° were obtained in the preoperative holding area. CT° was obtained using the 3M™SpotOn™ monitoring System (Model 370, 3M Science, St.Paul, MN, USA), where the single-use sensor was placed on the patient's forehead before surgery which provided a continuous noninvasive CT° monitoring. In this study, we refer to maternal hypothermia as core body temperature <36°C, according to the 2017 SIAARTI Best Clinical Practice on Perioperative Normothermia (28). Spinal anesthesia was performed with intrathecal isobaric 8 mg levobupivacaine and 20 μg fentanyl, administered *via* a 27-gauge Whitacre needle, inserted at the L2–L3 or L3–L4 intervertebral space. After the spinal anesthesia, patients were immediately positioned supine, and the uterus was manually displaced to the left. Intravenous crystalloid preloading (Ringer's lactate solution, 10 ml/kg) was infused 10 min before the lumbar puncture. Thereafter, a coload of 10 ml/kg/h Ringer's lactate solution was administered.

The WF group received the IV fluids coload warmed to 41°C through a 3M Ranger™ Fluid Warmer (Model 245, Arizant Healthcare, Maplewood, MN, USA). The AW group received WF, and a thermal blanket (forced-air warming system) was placed after spinal anesthesia all over the upper extremities and attached to the convective temperature management system 3M Bair Hugger™ (Model 505); the device was set to “high” (43°C) in the AW group and “ambient” in the WF and NW groups. NW group received IV fluid at room temperature (fluid warmer set to “off”), no active warming was administered, but routine care with blankets was provided.

### Room Temperature Was Measured in the Operative Room (OR) on the Arrival of Patients

Core temperature ° and mean arterial pressure (MAP) were recorded on the arrival of patient in the operating room (baseline), 5 min postspinal anesthesia (T5), every 10 min during surgery until the end of the cesarean delivery, and 30 min after the surgery (discharge).

Patients were also asked to report the severity of shivering using the Bedside Shivering Assessment Scale (BSAS) from 0 to 3 (0 = no shivering, 1 = shivering localized to the core and neck, 2 = shivering including the upper extremities, 3 = total body shivering) and the thermal comfort scores (TCS) obtained by using a visual analog scale from 0 to 100 (0 mm= worst imaginable cold, 50 mm = thermos-neutrality, and 100 mm = insufferably hot); these data were taken at 20 and 40 min (T20, T40) from baseline and at discharge from the OR.

Total volume of estimated blood losses during surgery was recorded at the end of surgery.

Apgar scores and axillary neonatal temperature were recorded at 1 and 5 min after birth, and umbilical vein BGA was obtained for analysis from a double-clamped umbilical cord.

### Statistical Analysis

The power analysis suggested that a sample size of 21 parturients/group was required to detect a 0.5°C difference among groups in maternal CT (assuming α = 0.01 and power = 0.95) (29). The number was then increased to 26 per group to allow for a 20% patients drop-out rate.

The normality of distribution was assessed by Shapiro–Wilkinson test. Since we found almost all of the data normally distributed, the data were expressed as mean ± SD values or number as appropriate. Data were analyzed using repeated measurements analysis of variance (RANOVA). Differences between the groups at each time point were examined *post hoc* using an independent sample *t*-test. A paired sample *t*-test was used to detect changes within the groups. Categorical data were analyzed by Chi-squared test (χ^2^). Level of statistical significance was chosen to be at *p* < 0.05. Statistical analysis was performed by Statistical Package for the Social Sciences (SPSS Inc., Chicago, IL, USA) version 15.0 for Windows.

## Results

**Figure 1:**
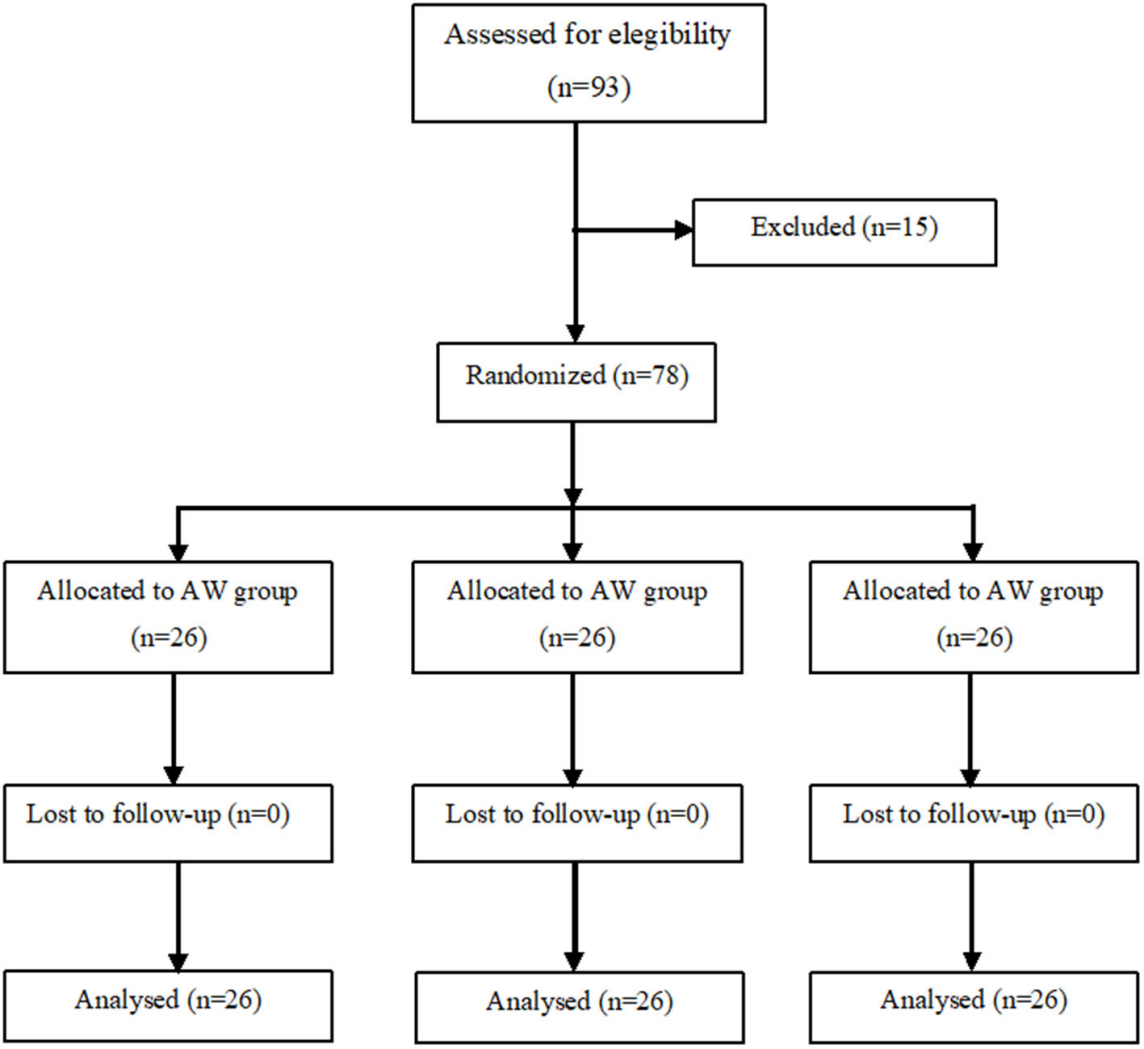
Flowchart of patient's enrolment.

We recruited 93 pregnant women of whom 78 were enrolled in the study and divided them into three groups of 26 patients each (Figure 1). There were no significant differences among the three groups as regards age, height, weight, BMI, gestational age, baseline temperature, and surgery duration (from surgical incision to skin closure) (Table 1).

**Table 1:** Demographic and obstetric data of the study population.

	**NW group (*n =* 26)**	**WF group (*n =* 26)**	**AW group (*n =* 26)**	***P***
Age (years)	34 ± 5	33 ± 7	36 ± 63	0.756
Height (cm)	163 ± 6	162 ± 6	163 ± 5	0.926
Weight (kg)	73 ± 11	75 ± 16	79± 14	0.227
BMI (kg/m^2^)	27 ± 3	28 ± 6	29 ± 5	0.194
Gestational age (weeks)	38 ± 7	38 ± 1	38 ± 1	0.205
Baseline CT° (°C)	36.9 ± 0.4	37 ± 0.4	37 ± 0.4	0.807
Duration of surgery (min)	52 ± 10	52 ± 10	48 ± 6	0.207
T°OR (°C)	23 ± 1	24 ± 2	23 ± 0.4	0.103

*Data are expressed in terms of mean ± standard deviation*.

*NW, IV fluids at room temperature and no forced-air warming; WF, warmed IV fluids; AW, warmed IV fluids and forced-air warming; P, p-value; CT, core temperature (°C); T°OR, temperature in the operating room (°C)*.

At the arrival of the patients, the operative room temperature (T°OR) was 23.4°C ± 1.4°C in the NW, 24.25°C ± 1.8°C in the WF group, and 22.5°C ± 0.4°C in the AW group, with no significant difference between the study groups.

Intergroup analysis showed that at baseline, the patient CT° was 36.9°C ± 0.4°C in the NW group; 37°C ± 0.5°C in the WF group, and 37°C ± 0.5°C in the AW group (*p* = 0.80).

At T25, the CT° of NW, WF, and AW groups were respectively 36.4°C ± 0.4°C, 36.6°C ± 0.3°C, and 36.8°C ± 0.3°C; CT° in AW group was significantly higher than WF group and NW group (AW vs. WF: *p* = 0.023; AW vs. NW: *p* = 0.001).

At T35, the CT° of NW, WF, and AW groups were respectively 36.3°C ± 0.5°C, 36.5°C ± 0.3°C, and 36.7°C ± 0.3°C; CT° in AW group was significantly higher than WF group and NW group (AW vs. WF: *p* = 0.025; AW vs. NW: *p* < 0.001). The CT° in WF group was also statistically higher than the NW group (*p* = 0.047).

At T45, the CT° of NW, WF, and AW groups were respectively 36.2°C ± 0.3°C, 36.5°C ±0.3°C, and 36.7°C ± 0.3; CT° in AW and WF groups was significantly higher than the NW group (AW vs. NW: *p* < 0.001; WF vs. NW: *p* = 0.004).

At discharge, the CT° of NW, WF, and AW groups were respectively 36.2°C ± 0.3°C, 36.4°C ± 0.3°C, and 36.7°C ± 0.3°C; CT° in the AW group was significantly higher than WF group and NW group (AW vs. WF: *P* = 0.001; AW vs. NW: *p* < 0.00).

**Figure 2:**
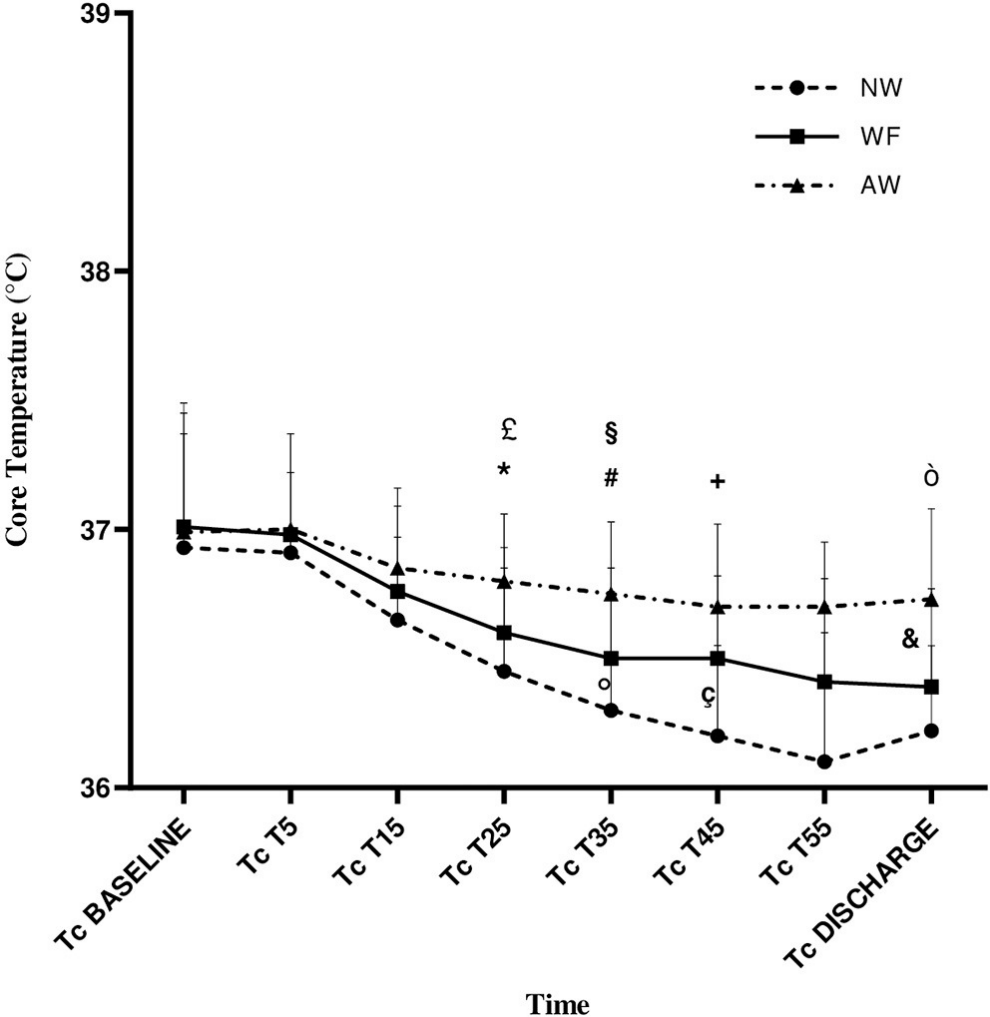
Core temperature (CT°) variation during the perioperative time. Data are expressed in terms of mean ± standard deviation. NW, IV fluids at room temperature and no forced-air warming; WF, warmed IV fluids; AW, warmed IV fluids and forced-air warming; P, p-value; CT°, core temperature, expressed in °C; T°OR, temperature in the operating room, expressed in °C; CT°, core temperature; T5, T15, T25, T35, T45, and T55: 5, 15, 25, 35, 45, and −55 min from baseline, respectively. £, AW vs. WF (p = 0.023); *, AW vs. NW (p = 0.001); #, AW vs WF (p = 0.025); §, AW vs NW (p < 0.001); °, WF vs NW (p = 0.047); +, AW vs NW (p < 0.001); ç, WF vs NW (p = 0.004); ò, AW vs. NW (p < 0.001); &, AW vs. WF (p = 0.001).

Intragroup analysis showed that the CT° gradually decreased in perioperative time in all the three groups and with significant difference from baseline to discharge (NW and WF: *p* < 0.001; AW: *p* = 0.045). CT° in our study was never <36°C in the AW group (Table 2, Figure 2).

**Table 2:** Maternal PH incidence.

**Outcome**	**NW group (*N =* 26) *n* (%)**	**WF group (*N =* 26) *n* (%)**	**AW group (*N =* 26) *n* (%)**	***P***
Peri-operative hypothermia (PH)	(47%)^*^^°^	1 (4%)^*^^#^	0 (0%)^#^^°^	0.04^*^
				0.31^#^
				0.009^°^

*Data are expressed in terms of % of women with PH for each study group*.

*PH, peri-operative hypothermia; NW, IV fluids at room temperature and no forced-air warming; WF, warmed IV fluids; AW, warmed IV fluids and forced-air warming; P, p-value*.

* *, NW vs WF (P = 0.04)*;

# *, WF vs AW (P = 0.31)*;

° *, NW vs AW (P = 0.009)*.

As regards the MAP, no differences were observed among the three groups during the study period.

Blood loss in perioperative time was 258 ± 80 ml in the NW, 218 ± 90 ml in the WF group, and 227 ± 55 ml in the AW group, with no significant difference between these groups.

**Figure 3:**
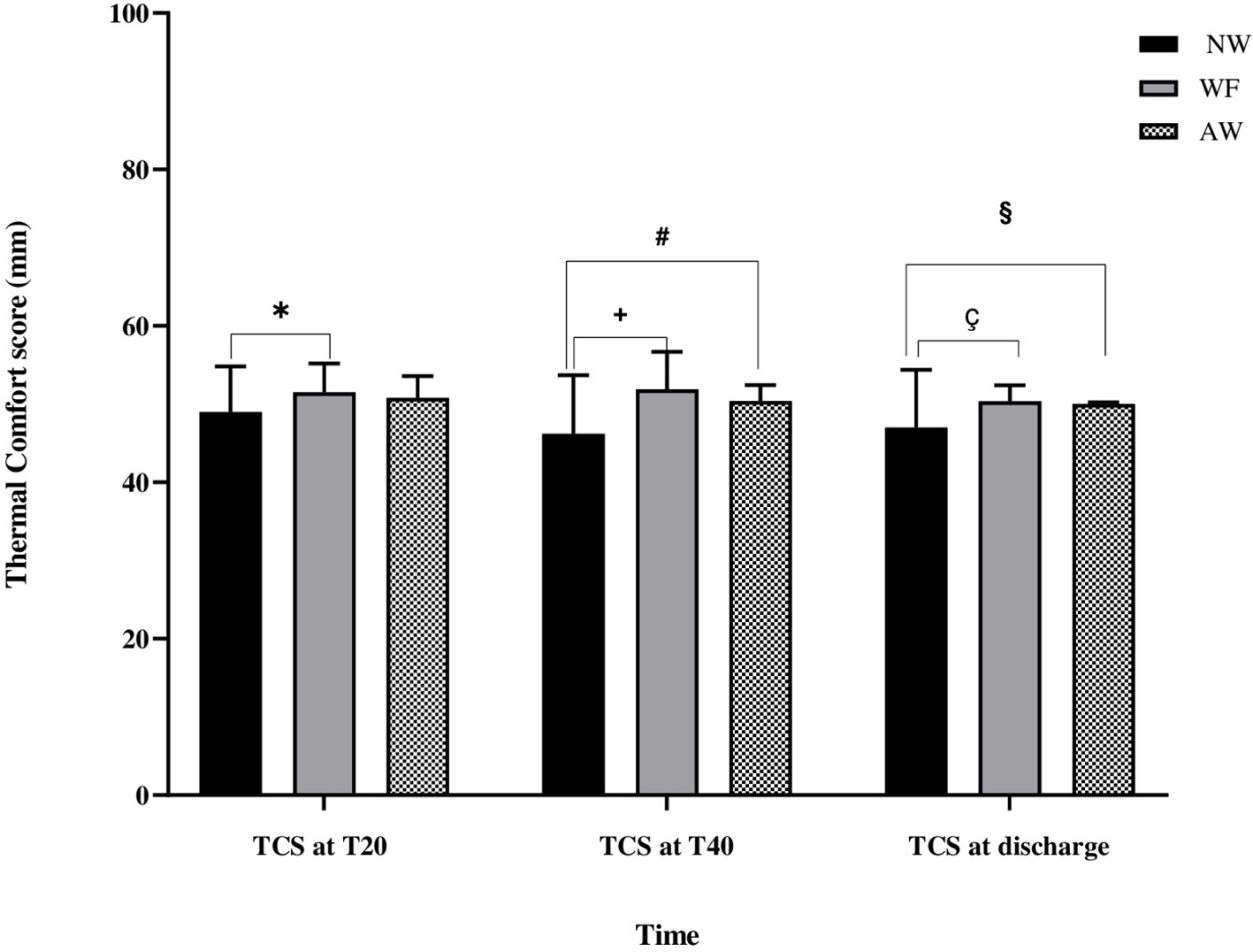
Thermal comfort score evaluation (TCS) during the perioperative time. Thermal comfort score evaluation. Data are expressed in terms of mean ± standard deviation. TCS, thermal comfort score (mm); NW, IV fluids at room temperature and no forced-air warming; WF, warmed IV fluids; AW, warmed IV fluids and forced-air warming; T20, at 20 min from baseline; T40, at 40 min from baseline. *, NW vs. WF (*p* = 0.04); #, NW vs. AW (*p* = 0.007); +, NW vs. WF (*p* < 0.001); §, NW vs. AW (p = 0.02); ç, NW vs. WF (*p* = 0.008).

The other two statistically relevant outcomes were the BSAS (Table 3) and the maternal TCS (Figure 3).

**Table 3:** Bedside shivering assessment scale evaluation (BSAS) during the perioperative time.

**BSAS time**	**score**	**NW group (*N =* 26) *n* (%)**	**WF group (*N =* 26) *n* (%)**	**AW group (*N =* 26) *n* (%)**	***P***
BSAS T20	0	22 (85%)^*^^+^	26 (100%)^*^	26 (100% ^+^	0.04^*^^+^
	1	1 (4%)	0 (0%)	0 (0%)	
	2	3 (11%)	0 (0%)	0 (0%)	
BSAS T40	0	12 (46%)^#^^ç^	24 (92%)^#^	26 (100%)^ç^	<0.001^#^^ç^
	1	10 (38%)	2 (8%)	0 (0%)	
	2	4 (16%)	0 (0%)	0 (0%)	
BSAS discharge	0	23 (88%)	25 (96%)	26 (100%)	0.383
	1	3 (12%)	1 (4%)	0 (0%)	
	2	0 (0%)	0 (0%)	0 (0%)	

*Bedside shivering assessment scale evaluation. Data are expressed in terms of mean ± standard deviation*.

*BSAS, bed shivering assessment scale; NW, IV fluids at room temperature and no forced-air warming; WF, warmed IV fluids; AW, warmed IV fluids and forced-air warming; T20, at 20 min from baseline; T40, at 40 min from baseline*.

* *, NW vs. WF (p = 0.04)*;

+ *, NW vs. AW (p = 0.04)*;

# *, NW vs. WF (p < 0.001)*;

ç *, NW vs. AW (p < 0.001)*.

At T20 and T40, the incidence of shivering was significantly higher in the NW than in the AW and WF groups (respectively, at T20: *p* = 0.04; at T40: *p* < 0.001).

At discharge, no shivering was observed. No patient had severe shivering (BSAS 3) in either group.

As far as thermal comfort is concerned, at T20, the TCS in the NW, WF, and AW groups were respectively 49 ± 6 mm, 51.5 ± 4 mm, and 51 ± 3 mm; TCS in NW group was lower only with respect to the WF group (NW vs. WF: *p* = 0.04).

At T40, the TCS in the NW, WF, and AW groups were respectively 46 ± 7 mm, 52 ± 5 mm, and 50 ± 2 mm; TCS in NW group was lower than WF and AW groups (NW vs WF: *p* < 0.001; NW vs. AW: *p* = 0.007).

At discharge, the TCS in the NW, WF, and AW groups were respectively 47 ± 7 mm, 50 ± 2 mm, and 50 ± 0 mm; TCS in NW group was lower than WF and AW groups (NW vs. WF: *p* = 0.008; NW vs. AW: *p* = 0.02). No patient reported insufferable hot during warming.

There were no significant differences among the three groups in terms of APGAR score and neonatal body temperature at 1 and 5 min from birth, birth weight, and umbilical cord vein BGA (Table 4).

**Table 4:** Neonatal demographics, APGAR scores, umbilical cord vein BGA, and temperature data.

	**NW group** **(*N =* 26)**	**WF group** **(*N =* 26)**	**AW group** **(*N =* 26)**	***P***
APGAR score 1 m	8 ± 8	8 ± 5	8 ± 3	0.948
APGAR score 5 m	8.9 ± 2	8.9 ± 3	9 ± 1	0.099
Temperature (°C) 1 m	35.9 ± 4	35 ± 7	36.6 ± 2	0.430
Temperature (°C) 5 m	35.9 ± 3	34.9 ± 7	36.6 ± 2	0.364
Birth weight (g)	2,966 ± 433	3,245 ± 597	3,087 ± 314	0.104
Umbilical cord vein BGA	7.32 ± 0.05	7.34 ± 0.05	7.33 ± 0.06	0.454

*Data are expressed in terms of mean ± standard deviation*.

*NW, IV fluids at room temperature and no forced-air warming; WF, warmed IV fluids; AW, warmed IV fluids and forced-air warming; p, p-value; BGA, blood gas analysis*.

No infant had complications or died.

## Discussion

The main results of our study demonstrated that the combination of AW is effective in reducing the loss of maternal CT° and the incidence of shivering during cesarean delivery (CD) in patients undergoing spinal anesthesia.

Warmed IV fluids, when used alone, are useful in maintaining a higher temperature during surgery and in postoperative time compared with no use of warming systems (NW), but they provided the worst results, in terms of incidence of shivering, compared with AW.

Perioperative hypothermia (PH), defined as a CT below 36°C, is one of the most common phenomena in surgical patients (30). Hypothermia during surgery can be generated by various factors, such as exposure to the surgical environment, thermoregulatory dysfunction, and medications.

The primary mechanism of perioperative hypothermia during CD, after neuraxial anesthesia, is the redistribution of intravascular volume from the core to the peripheral compartment. Moreover, after a drop of CT, hypothermia triggers vasoconstriction and shivering in unblocked regions.

Perioperative hypothermia leads to intraoperative blood loss, cardiac events, coagulopathy, an increase in hospital stay and associated costs (31). Excluding therapeutic uses of hypothermia, such as in cardiac arrest patients and neonatal hypoxic-ischemic encephalopathy, perioperative PH should be avoided (32, 33).

Currently, a plethora of patient warming devices is utilized to reduce the incidence of intraoperative hypothermia; the most used are forced-air warming systems, which warm the patient from the outside, and WF, which may prevent a decrease in body temperature in the setting of redistribution hypothermia (3). Several studies have shown that WF, as a single modality of warming, are effective in minimizing maternal PH, but did not reduce the incidence of shivering (23, 31, 34, 35).

In other studies, forced-air warming was used as the only warming method. Butwick et al. applied the forced-air warming on the lower extremities to warm the peripheral compartment below the level of sympathetic inhibition, but they found no improvements in preventing PH and shivering (4). Fallis and Horn et al. used forced-air warming on the upper extremities, but they obtained contrasting results in terms of maternal CT° and shivering (20, 21). Therefore, the optimal area of application (upper or lower) for forced-air warming still needs be determined.

In the last few years, many groups focused their study on the effects of combining AW on maternal CT° and shivering incidence.

Cobb et al. used combined intraoperative WF and lower body forced-air warming, and they found good results compared with no warmed control group in reducing the trend of maternal CT° decline and greater maternal thermal comfort, but they still observed high incidence of PH and no decrease in shivering incidence (2).

In contrast with our study, patients were hypothermic in AW group. A potential explanation could be that our surgeons were faster in performing CD.

In regards to the timing of application of warming systems, some studies demonstrated that prewarming is effective in preventing redistribution hypothermia after both neuraxial and general anesthesia (20, 36).

With the aim of reducing redistribution hypothermia and core-to-periphery temperature gradient, Jun et al. treated patients with forced-air warming placed over the entire body 15 min before starting anesthesia and WF coload during surgery (11). This strategy yielded only little efficacy in preventing perioperative CT° decline and shivering in the warmed group with respect to no warmed control. Our work demonstrated that the combination of AW was effective when active warming interventions are applied during the intraoperative time, when forced-air warming started soon after the spinal anesthesia and continued for the whole time of surgery.

Horn et al. applied forced-air warming on the upper extremities 15 min before anesthesia, showing an additional efficacy when it was combined with WF in the setting of epidural anesthesia and CD (20). Differently, we applied forced-air warming after spinal anesthesia.

Furthermore, Meghana et al. compared the use of combined lower extremities AW to only forced-air warming modality. They observed that combined warming methods preserved maternal CT° and reduced shivering in patients undergoing CD and spinal anesthesia, recommending the use of active warming methods in the operation theater (22).

In our work, the combined AW interventions dampened the decline in CT° in perioperative time and decreased the incidence of shivering more than WF group, although the difference was not significant, and we cannot make a strong suggestion for AW strategy as routine care during CD.

As far as concern blood losses during surgery, a recent metaanalysis indicates that even mild hypothermia (CT° 34–36°C) increases blood loss and the relative risk of transfusion by small but significant amounts compared with normothermic surgical patients (37, 38). In our work, we did not observe a significant difference in blood loss among the three study groups.

Previous studies used infrared tympanic thermometer or oral probes whose reliability has been questioned (39). In our work, we used the SpotOn which is a new noninvasive, skin temperature monitoring system that measures the heat-flux in temporal artery by using a single-use sensor placed on the patient's forehead. Indeed, the SpotOn is much more sensitive to temperature changes when compared with other noninvasive devices.

In this work, our primary goals were the evaluation of maternal CT°, the incidence of shivering, and the maternal thermal comfort in perioperative period for women undergoing CD.

Secondary goals included blood loss evaluation and neonatal outcomes (Apgar score, axillary temperature, birth weight, and umbilical cord vein BGA), neither of them showing significant variation; these latter results are not surprising given our limited measures of neonatal well-being.

One potential limitation of our study consisted of the use of a nonvalidated visual analog scale for thermal comfort quantification. We used the same scale devised by Horn et al. (20) (0 mm = worst imaginable cold, 50 mm = thermoneutrality, and 100 mm = insufferable hot), which was not validated and was a little confusing to patients. Nevertheless, this scale can well differentiate between the hot and cold spectrum of discomfort compared with the one used by other studies that indicate with a verbal numerical scale the thermal comfort in terms of maternal satisfaction (0 = completely unsatisfied and 100 = completely satisfied) (2, 11).

Another limitation consisted of newborn CT° measured with axillary thermometer rather than the SpotOn system, which has a low level of accuracy as a method of monitoring body CT° in pediatric patients (<10 kg) (40).

## Conclusions

In summary, our study showed that the combination of AW systems for elective CD under spinal anesthesia increases maternal CT°, prevents PH, and reduces the overall perioperative CT° decline and the incidence of shivering compared with the use of only WF or no interventions.

Based on the study findings, identification of women at risk of developing perioperative hypothermia may allow for more targeted AW strategies.

## Data Availability Statement

The raw data supporting the conclusions of this article will be made available by the authors, without undue reservation.

## Ethics Statement

The studies involving human participants were reviewed and approved by Comitato Etico Area 1-Azienda Ospedaliera Riuniti di Foggia (DDG n. 363 del 25.10.2016 e s.m.i. DDG n. 318 del 14.6.2019). The patients/participants provided their written informed consent to participate in this study.

## Author Contributions

AC was responsible for the conception and design of the study, analyzed the data of the study, and wrote the manuscript. PM analyzed the data and wrote the manuscript. CF organized the database. PD performed the study. RB and SZ wrote a section of the manuscript. GC revised the final manuscript. AC, PM, CF, PD, RB, SZ, and GC approved the final manuscript. All authors contributed to the article and approved the submitted version.

## Conflict of Interest

The authors declare that the research was conducted in the absence of any commercial or financial relationships that could be construed as a potential conflict of interest.

## Publisher's Note

All claims expressed in this article are solely those of the authors and do not necessarily represent those of their affiliated organizations, or those of the publisher, the editors and the reviewers. Any product that may be evaluated in this article, or claim that may be made by its manufacturer, is not guaranteed or endorsed by the publisher.

## Abbreviations

ASA: American society of anesthesiologists
AW: warmed IV fluids and forced-air warming
BGA: blood gas analysis
BMI: body mass index
BSAS: bedside shivering assessment scale
CD: cesarean delivery
CT°: core temperature (°C)
MAP: mean arterial pressure
NW: no warming
OR: operating room
P: p-value
PAC: pulmonary artery catheter
PH: perioperative hypothermia
SD: standard deviation
T° OR: temperature in the operating room
T5-15-20-25-35-40-45-55: time at 5-15-20-25-35-40-45-55 min from baseline
TCS: thermal comfort score
WF: warmed IV fluids.
